# Japan’s Powerful Ally

**Published:** 1913-08

**Authors:** 


### Abstracts and Selections.

### Japan's Powerful Ally.

If statistics count for anything, in a war between Japan and the United States, Japan could count on an ally about thirteen times as powerful as herself. The name of this ally is Disease, which in the Spanish-American war struck down thirteen of our men to every one that fell from the enemy's bullets, whereas in the Russo-Japanese war the Japanese lost only 21,559 by disease to 55,679 in battle. The co-operation of disease with our enemy would of course be dependent on ourselves. If our army officers tackle the problem as they did the fever scourge in Panama, we have nothing to fear—and yet our army included Colonel Gorgas and others as skilled when our men were dying off by thousands at the Chickamauga camp. In time of war, science often abdicates, and another leader, known as politics, takes the reins. If we are ever going to fight Japan, or any other nation, we must alter this probability. There has been some effort of late to belittle Japan's achievements in sanitation during her war with Russia. Dr. Louis Livingston Seaman, who first brought the reports of Japanese army sanitation to this country, and is still the best authority on the subject, writes in the *New York Times* (June 22) that these efforts are beside the mark. The final medical statistics of the war, just issued, show that the Russian sanitary work was also noteworthy, their army losing 12,128 from disease, and 31,458 from wounds, showing a total loss of only 43,-586 against Japan's 77,238. Our own record in our next war may be equally good, but we have no past performances to show for it. Says Dr. Seaman:

"The astonishing fact revealed in the figures for the Japanese is that out of 77,238 dead, 55,679 met death from battle casualties, leaving 21,559 who died from all other causes together. * * * While the ratio for the Russians is about the same, it still must be borne in mind that the Japanese were the aggressive foe, fighting in the open against an enemy intrenched in his mountain and other strongholds, and, further, that the severity of the wounds sustained by the Japanese was accentuated by the difference in caliber of the rifles used by the Russians.

"Compare this wonderful achievement of the Japanese with Longmore's Tables, based on the records of battles for the last two hundred years, which are accepted as the most reliable statistics of war, and which show that rarely has there been a conflict of any great duration in which at least four men have not perished from disease to every one from bullets. Yet the Japanese lost nearly three men from battle casualties for every one man from disease.

"In the Russo-Turkish War 80,000 men died from disease and 20,000 from wounds. It is asserted by eminent authority that in six months of the Crimean campaign the allied forces lost 50,000 from disease and but 20,000 from bullets. A gentleman who remembers that campaign, an ex-President of the New York Academy of Music, told me that he had seen whole regiments die from disease without ever reaching the firing line.

"In our war with Mexico the proportion of losses was about three from disease to one from bullets, and in our great Civil War nearly the same proportion obtained. In round numbers, of the hundreds of thousands of fatalities in that conflict, nearly three-quarters resulted from disease. Almost as many men perished from fevers and intestinal diseases as were slaughtered in the terrible battles that ended our great conflict.

"No lessons seem to have been learned from these frightful experiences, for later statistics show no improvement. In the French campaign in Madagascar, in 1894, 14,000 men were sent to the front, of whom 29 were killed in action and 7000 perished from preventable diseases. In the Boer War in South Africa the English losses from disease were simply frightful, greater even than our Civil War.

"But the crowning piece of imbecility was reserved for our war with Spain, when in 1898 more than thirteen men were needlessly sacrified to ignorance and incompetency for every one who died on the firing line or from battle casualties, and that, too, in a war the chief campaign of which lasted only six weeks.

"Without for a moment minimizing the splendor of her victories on land and sea—Mukden, Port Arthur, Liaoyang, and the Korean Straits, of which two are among the bloodiest battles of history—I still assert unhesitatingly that the greatest conquests of Japan have been in the humanities of war, in the stopping of needless sacrifice of life by the prevention of disease."

Long before the opening of hostilities, Dr. Seaman tells us, thorough preparations had been made in the hospital service, it being recognized that every soldier saved was a double gain. Japan made the medical department of her army of equal importance with that of the fighting branch, and ranked its officers accordingly. The Japanese were the first to recognize the true value of the medical officer in time of war. He goes on:

"In her record-breaking campaign he was given an auxiliary force of more than 44,000 men, known as 'sanitary soldiers'—an absolutely unknown factor in our army. These sanitary soldiers were subservient to the medical officers—to carry out sanitary regulations—serve as hospital stewards, litter carriers, or in any other capacity to which he might detail them. And throughout the way they proved a most powerful factor in the fight against preventable disease.

"The medical officer or one of his sanitary soldiers was omnipresent in the Russo-Japanese War, just as he would be today should another conflict arise. He was to be found in countless places where in an American or a British Army he has no place. He was as much at the front as at the rear. He was with the first screen of scouts, testing and labeling wells or procuring samples of water, so that the army to follow should not drink contaminated water. When the scouts reached a town he immediately started a thorough examination of its sanitary condition, and if contagion or infection was found, he quarantined and placed a guard around the dangerous district.

"Microscopic blood tests were made in all fever cases, and bacteriological experts, fully equipped, formed part of the staff of every divisional headquarters. Japan recognized that microscopes were as important as eleven-inch guns, and she sent these experts to the front, where they found their way into every place where an extended stay was made and where bacteria were likely to be found. The medical officer, or sanitary soldier, also accompanied the foraging parties, and, with the commissariat officers, sampled food, fruit and vegetables sold by the natives along the line of march long before the arrival of the army. If the food was found tainted or overripe, of the water required boiling, notice was posted to that effect; and such was the discipline of every soldier, from commanding officer to the rank and file, that obedience to that order was absolute.

"The medical officer was also found in the camp, lecturing the men on sanitation and the hundred and one details of personal hygiene—how to cook and eat, when not to drink or bathe—even to the paring of the finger nails to prevent danger from bacteria. In their surgical work the Japanese were extremely conservative. The scalpel was rarely in evidence, and amputations were comparatively few, both in the arm yand navy. * * *

"The splendidly systematized equipment of the pharmaceutical department was an instructive example from which Occidental nations might well profit. Shortage of ammunition in the emergencies of battle sometimes occurred, but never was there any deficiency of medical supplies, as I personally observed, and as did every other writer and surgeon I met.

"Even during the battle of Mukden, which lasted sixteen days, and in that at Liaoyang, every hospital in operation was well supplied with medical and surgical necessities. Every soldier had his first-aid package and knew how to use it. No operations were permitted on the field, except in cases of emergency where death was imminent from hemorrhage, reliance being placed on the efficiency of the aseptic first-aid dressing."

Supplementing this magnificent work in the field, Japan organized at home the most splendid system of hospitals that had ever been devised, and put into execution the most elaborate and effective system of sanitation that had ever been practiced in war. On the declaration of war, she was prepared to house, treat, and care for 25,000 sick and wounded in Japan alone. These accommodations were rapidly increased, until a year and a half later, the great military hospitals possessed a normal capacity of 58,-263 available beds. That this was necessary was demonstrated after the battle of Mukden, when the total hospital capacity of 80,000 beds was taxed to its limits by the shattered men who poured in by the thousands from every transport. Dr. Seaman then brings the lesson home as follows:

"Such are some of the lessons taught by the Japanese in the art of preparation, and, thank heaven, they have not been wasted on the American army. Another instance may be cited here of the perfection of preparation and detail exercised by the medical department of the Japanese. When two armies faced each other for mortal combat in Mongolia—with one hundred miles of firing-line, awaiting the outcome of the battle, which, it was predicted, would throw the bloody record of Mukden in the shade—62,000 clean, empty beds stood awaiting with plenty of attendants, ready to receive the wounded, who never came.

"Compare with this those infected pest holes, Camp Black, Chickamauga, Camp Alger, in sight of the Capitol's dome, or Montauk Point, which awaited the sick and wounded of our army four months after the battle of San Juan, and then let critics assert we have nothing to learn from the Japanese—and that their sanitation was a 'myth.'"—*Literary Digest,* July 19.

